# Reproducibility of postural control measurement during unstable sitting in low back pain patients

**DOI:** 10.1186/1471-2474-8-44

**Published:** 2007-05-22

**Authors:** Ulrike Van Daele, Stefanie Huyvaert, Friso Hagman, William Duquet, Bart Van Gheluwe, Peter Vaes

**Affiliations:** 1 Institute of Physical Therapy and Occupational Therapy, Department of Health Care, University College of Antwerp, Belgium; 2 Faculty of Physical Education and Physical Therapy, Vrije Universiteit Brussel, Belgium

## Abstract

**Background:**

Postural control tests like standing and sitting stabilometry are widely used to evaluate neuromuscular control related to trunk balance in low back pain patients. Chronic low back pain patients have lesser postural control compared to healthy subjects. Few studies have assessed the reproducibility of the centre of pressure deviations and to our knowledge no studies have investigated the reproducibility of three-dimensional kinematics of postural control tests in a low back pain population. Therefore the aim of this study was to assess the test-retest reproducibility of a seated postural control test in low back pain patients.

**Methods:**

Postural control in low back pain patients was registered by a three dimensional motion analysis system combined with a force plate. Sixteen chronic low back pain patients having complaints for at least six months, were included based on specific clinical criteria. Every subject performed 4 postural control tests. Every test was repeated 4 times and lasted 40 seconds. The force plate registered the deviations of the centre of pressure. A Vicon-612-datastation, equipped with 7 infra-red M1 camera's, was used to track 13 markers attached to the torso and pelvis in order to estimate their angular displacement in the 3 cardinal planes.

**Results:**

All Intraclass Correlation Coefficients (ICC) calculated for the force plate variables did not exceed 0.73 (ranging between 0.11 and 0.73). As for the torso, ICC's of the mean flexion-extension and rotation angles ranged from 0.65 to 0.93 and of the mean lateral flexion angle from 0.50 to 0.67. For the pelvis the ICC of the mean flexion-extension angle varied between 0.66 and 0.83, the mean lateral flexion angle between 0.16 and 0.81 and the mean rotation angle between 0.40 and 0.62.

Consecutive data suggest that the low test-retest reproducibility is probably due to a learning effect.

**Conclusion:**

The test-retest reproducibility of these postural control tests in an unstable sitting position can globally be considered as rather moderate. In order to improve the test-retest reproducibility, a learning period may be advisable at the beginning of the test.

## Background

Postural control variables have often been used for evaluating patients with various neuromuscular disorders. Several recent studies indicated that patients with low back pain (LBP) show poorer postural control standing on a force plate compared to healthy controls [1-6]. Cholewicki and co-workers used a postural control task in an unstable sitting position. In standing, postural adjustments can be accomplished with a wide range of responses through the ankle, knee, hip, and lumbar spine joints. In contrast, the postural control of the lumbar spine, while seated, is isolated from the control of lower extremity joints [1,7]. In the study of Cholewicki and co-workers four levels of seat instability were introduced by using smaller diameters for the hemisphere that were attached below the seat surface (infinity and radii of 50, 44 and 22 cm). Displacements of the centre of pressure (CoP) underneath the seat were measured with a force plate. Patients with low back pain had poorer balance performance than the healthy control group, especially for the most difficult level (diameter = 22 cm). They also found a correlation between impaired postural control and delayed muscle response time (measured by surface electromyography of 12 major trunk muscles) for patients with LBP. This suggests a common underlying pathology in the lumbar spine being responsible for the impaired postural control [1,7].

Research has shown that the process of maintaining or regaining postural stability requires considerable information-processing [8-16]. Therefore, the control of erect posture may be more integrated into the movement control scheme than has been previously considered [6]. Integrated information from three independent sensory sources (somatosensory, vestibular and visual) are used to maintain postural stability. To control and maintain body-balance this information is constantly evaluated and adjusted. Disturbance of any one of the three sensory systems will influence the overall output of the postural system [17]. Since only somatosensory input is of interest here, we choose to blindfold the subjects and to exclude any person with vestibular dysfunctions.

When postural control is used as an evaluation tool, test-retest reproducibility is an important factor. Intraclass Correlation Coefficients (ICC's) of variables of Center of Pressure (CoP) displacements were determined in three studies. Nies-Byl et al. found high ICC values between 0.82 and 0.92 [18], others found moderate to low ICC values with a wide range between 0.1 and 0.7 [19], still others found ICC's between 0.4 and 0.6 [20].

Beside through CoP displacements, postural control can also be evaluated using a three-dimensional motion analysis. Most of the research studied CoP displacements [1-7,18-20]. Only Mientjes et al. [20] and O'Sullivan et al. [21] performed a three- dimensional motion analysis. A three-dimensional motion analysis of a postural control test in unstable sitting position offers more information about the difference in balance strategies. Mientjes et al. collected kinematic data only in the anterior-posterior direction. Because these data showed a broad range in the responses of the individual chronic LBP patients the test-retest reproducibility of the kinematic data was not investigated in this study [20]. Therefore the goal of the present study is to investigate the test-retest reproducibility of a postural control test, with and without arm movement, in an unstable sitting position in low back pain patients. Hereby three-dimensional motion analysis was used in combination with force plate measurements.

## Methods

### Subjects

The selection of LBP patients was based on clinical criteria.

Inclusion criteria were: having at least 6 months of LBP and having at least two episodes of acute low back pain, having consulted at least once a medical doctor because of their LBP, have no neurological symptoms, have a visual analogue score for pain not higher than 60 and receiving physical therapy.

Exclusion criteria were obesity (body mass index > 30), vestibular dysfunction, vertebral fractures, muscle- nerve- skin- or joint diseases, scoliosis or kyphosis and pregnancy.

16 LBP patients, six men and ten women, were included. The mean age of the 16 subjects was 33 years ± 12 years, the mean weight was 71 kg ± 15 kg, the mean height was 170 cm ± 11 cm. The mean visual analogue score for pain was 17.56/100 ± 17.61. The mean Quebec Back Pain Disability Score was 23.2/100 ± 13.2 [22].

The study was approved by the Ethics Committee of the Academic Hospital of the Vrije Universiteit Brussel. Informed consent was signed by every participant.

### Measurement procedures

A Kistler force plate (60 cm × 90 cm) registered the deviations of the CoP and was time synchronised with the Vicon motion analysis system.

The three-dimensional motion analysis system consisted of the Vicon612-datastation equipped with the 7 M1 cameras and appropriate software. To track trunk and pelvis movement, 13 reflecting markers with a diameter of 24 mm were used. Using palpation methods these were attached on the processus spinosus of C7, T7, L3, the dorsal corner of the acromion (left and right), the highest part of the crista iliaca (left and right), the PSIS (left and right), the tuberositas tibiae (left and right), and the lateral malleoli (left and right). All data was sampled at 60 Hz.

A Kistler force plate (60 cm × 90 cm) registered the deviations of the CoP and was time synchronised with the Vicon motion analysis system.

The three-dimensional motion analysis system consisted of the Vicon612-datastation equipped with the 7 M1 cameras and appropriate software. To track trunk and pelvis movement, 13 reflecting markers with a diameter of 24 mm were used. Using palpation methods these were attached on the spinal process of C7, T7, L3, the dorsal corner of the acromion (left and right), the highest part of the iliac crests (left and right), the PSIS (left and right), the tibial tuberosities (left and right), and the lateral malleoli (left and right). All data was sampled at 60 Hz.

Technical description of palpation methods used to attach the markers:

In standing position:

The dorsal corner of the acromion is palpated.

C7: the last cervical spinal processes are palpated with the head in flexion. The subject performs a head extension, spinal process of C6 disappears, spinal process of C7 stays prominent.

T7: Spinal process located at the same height as the inferior angle of the scapula.

The subjects takes place on the wobble board:

The middle part of both tibial tuberosities are palpated.

The most lateral part of the lateral malleoli are palpated.

The highest part of the iliac crest is palpated.

Using the tumbs the researcher palpates the PSIS, he asks the subject to perform a hip flexion and extension.

Starting at the sacrum between the PSIS the researcher palpates upwards to find the fifth lumbar vertebra. He palpates upwards to the spinal process of L4 and L3. A marker is attached to the spinal process of L3.

### Tests

Subjects were blindfolded and sat on the flat side of a wobble board (board diameter: 43 cm, diameter hemisphere: 10 cm, height hemisphere: 5 cm). The wobble board was placed on a crutch, the height of which could be adjusted if necessary. The subjects sat with their hips and knees 90° flexed to place the wobble board in a horizontally balanced position.

**Figure 1:**
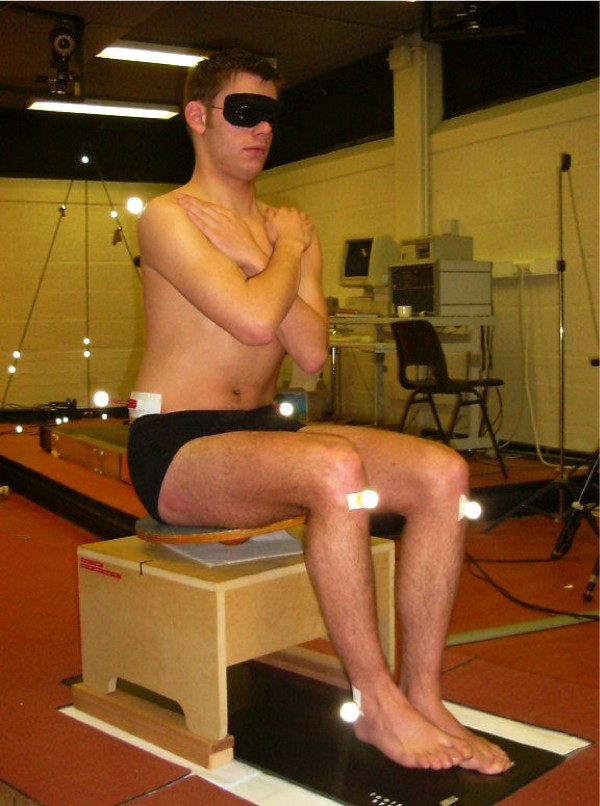
Test position 1.

**Figure 2:**
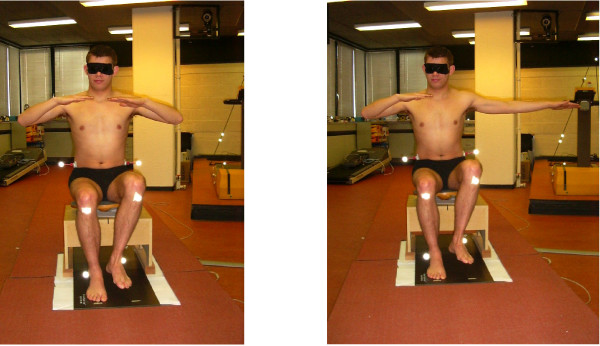
Test position 4.

All participants were subjected to 4 postural control tests. Before starting the test a static reference registration was taken during 5 seconds for calibration purposes. During the first test the subject sat with straight back, arms crossed in front of the chest, hands resting on the shoulders (figure 1). The measurement started when the subject was instructed to lift the right foot. The subject had to maintain his equilibrium during 40 seconds and was instructed to sit as still as possible during this time. Three trials were registered. In the second postural control test the left foot had to be lifted. Again three trials of 40 seconds were performed. In the third postural control test the subjects were instructed to flex the arms in front of the chest, fingers toward each other, elbows pointing to the sides. The measurement started when the subject lifted the right foot and then extended and flexed the arms to the sides, one arm at the time. Again three trials of 40 seconds were registered. In the fourth postural control test the same procedure was repeated with the left foot lifted (figure 2). After 12 trials (3 trials in each of the 4 different test positions) the blindfold was removed.

After a ten minute break the subject was repositioned on the wobble board and a fourth trial of 40 seconds was repeated for every starting position in the same order as the first time.

### Variables

#### Force plate

For each trial the mean application point of the CoP during the 40 second test was determined. The maximal, range, mean and standard deviation of the CoP deviations (in mm) from this mean application point were calculated. The same variables were calculated for the velocity of the CoP deviation (in mm/sec).

#### Three-dimensional motion analysis

The movement of pelvis and torso were analysed. The pelvis segment was defined by the two markers on the crista iliaca and the midpoint between the markers on the PSIS. Specifically, the line through the midpoints of the crista iliaca markers and the PSIS markers was used to define its axes in the saggital plane (direction posterior-anterior). The axes in the frontal plane was in the plane defined by the markers and perpendicular to the saggital plane. The axes in the transverse plane was constructed such that a right-turning orthogonal reference frame was defined for the pelvis.

The torso segment was defined by the markers on the left and right acromion and the midpoint between left and right crista iliaca. The local reference frame for the torso was constructed similar to the reference frame of the pelvis.

Angular data in all three directions: flexion-extension (F/E), left and right rotation (LR/RR), left and right latero-flexion (LL/RL), were calculated using the Cardan angle method with the order of rotations being: (1) F/E, (2) LL/RL, and (3) LR/RR.

In all these directions the mean angle and angular velocity was calculated.

Before calculations were performed on the marker data, individual marker paths were filtered using a low-pass second order recursive Butterworth filter with inverse padding; cut-off frequency was set to 20 Hz and 10-points of padding were used.

### Statistics

The Kolmogorov-Smirnov test was used to check normal distribution of all the variables (p < .001). The test-retest reproducibility was evaluated with the intraclass correlation coefficient (mixed model for absolute agreement). SPSS 14.0 for Windows was used for all statistics. Significance thresholds were set at .05.

## Results

The three-dimensional movement data of one person was excluded from the statistical analysis because of artefacts reducing the number of subjects to 15 for the statistical analysis of pelvis and torso motion. Force plate data were complete implying the contribution of all 16 subjects.

**Figure 3:**
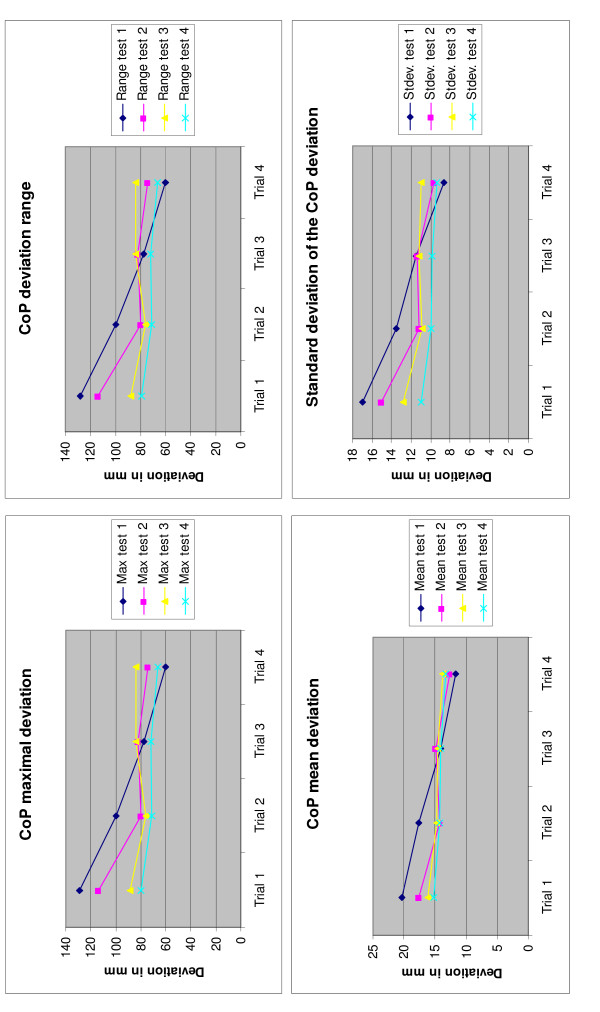
Mean values of maximal, range, mean and standard deviation of the CoP deviation distance to the mean CoP application point is shown for each trial and each test.

Figure 3 shows the population means of the maximal, the range, the mean and the standard deviations of the CoP deviations from the mean point of CoP application for each trial and each test. Figure 3 shows decreasing values from the first to the last trial for the variables mentioned above. The steepest decrease is shown in all four variables of test 1. A marked difference in means of postural control test 1 and 2 is shown in graphics of all variables.

The ICC's between the 4 consecutive trials for the CoP variables in 4 different postural control tests range from 0.11 to 0.73 (table 1).

For the mean CoP distance and the standard deviation of CoP distance the standard error of measurement (SEM) range between 3.12 mm and 7.11 mm. For the maximal CoP distance and range of CoP distance the SEM range between 37.40 mm and 51.77 mm. SEM from the mean deviation velocity and standard deviation of CoP velocity range between 17.35 mm/sec and 40.32 mm/sec. SEM from the maximal and range CoP velocity range between 285.57 mm/sec and 414.73 mm/sec.

As for the three-dimensional motion variables for the torso the ICC's of the mean flexion-extension angles range between 0.65 and 0.93, those of the mean rotation angle between 0.80 and 0.82 and those of the mean lateral flexion angle between 0.49 and 0.67 (table 2). SEM from these variables range between 1.3° and 4.4°.

As for the movement of the pelvis the ICC's of the mean anterior-posterior angle range from 0.66 to 0.83, those of the mean lateral flexion angle from 0.16 to 0.81 and of the mean rotation angle from 0.43 to 0.62 (table 3). SEM from these variables range between 1.8° and 4.3°.

ICC's of the mean angle velocities are in general very low (table 4 and 5). SEM of these variables range between 0.1°/sec and 0.4°/sec.

## Discussion

In table 1 to 5 all the ICC's show a large range. For the CoP variables the ICC range is comparable with the results of Takala et al. (ICC range 0.1–0.7) [19] and the results of Mientjes et al. (ICC range 0.4–0.6) [20]. Using the qualitative interpretation scale of ICC's as proposed by Landis and Koch [23], these reliability scores range from slight (ICC between 0–.20) to barely substantial (ICC between 0.61 and 0.80). Remarkable is that the ICC's of the mean CoP deviations and mean velocities are substantial in almost all the postural tests and that the ICC's of the maximal value, range or standard deviations are low. The low test-retest reproducibility could be explained by the search for better control strategies to maintain the equilibrium. The subjects were not allowed to practise before starting the actual test. Furthermore there is a remarkable trend, although not significant, in the results that show a systematic decrease of the deviations of postural control measured by the force plate, even the standard deviation decreases (see figure 3). This decrease is the most remarkable in the mean values of trials 1,2 and 3 of the first postural control test and the mean value of trial 1 of the second postural control test. This suggests a learning effect at the beginning of the test procedure. ICC's from CoP deviations were therefore recalculated using only trials 2, 3 and 4. Only the ICC's of postural test 2 were remarkably higher after this recalculation (0.66–0.74). It seems that the learning effect tends to stabilise after performing the trials of the 1st postural control test and the first trial of test 2. The only difference between postural control test 1 and 2 is the side of the lifted foot, a factor which can not be assumed to be responsible for the observed decreases. The fatigue factor can also not be held responsible for the observed decreases as a growing fatigue would rather lead to an increase of CoP deviations, which is not the case. The only remaining plausible factor seems to be a learning effect.

The studies describing test-retest reproducibility of postural control measurements in low back pain patients did not mention or investigate the possibility of a learning effect being the explication for the moderate ICC values [19,20]. Postural control studies in other populations investigating test-retest reproducibility have described the presence of a learning effect. A protocol designed for testing postural control in narrow spaces showed a learning effect of repeated static stabilometry in four groups of healthy subjects. First, the subjects were asked to stand on a bare platform with the eyes open, thereafter with the eyes closed. This was repeated with a foam rubber mat placed on top of the balance platform. Every test was repeated 10 times. The time interval between the first and the last test sequence was 11 (10–13) days for the test subjects in group I (n = 22), 17 days for group II (n = 13), 31(28–36) days for group III (n = 15) and 115 (49–193) days for group IV (n = 10).The learning effect was largest when standing on a foam rubber mat with eyes closed and when the time intervals between the tests were shortest. There was no difference in sway pattern or learning ability between tall and short test subjects, between subjects with heavy and light body weight or between the sexes [24]. In an other eyes-closed postural control test, proprioceptive information was altered by rotating the support surface. When the support surface returned to a level orientation, most subjects developed a body sway oscillation that differed significantly from the low-amplitude body sway typically observed during quiet stance. Oscillatory behaviour declined with increasing repetition of trials, suggesting a learning effect [25]. An other study showed that postural sway during unipedal stance with eyes closed increases lesser compared to bipedal stance with eyes open in a group of gymnasts than in a group of experts in other sports[26]. Gymnasts can be considered as experts in balance exercise compared to the other individuals active in sports. We could conclude that the experience of the gymnasts in balance strategies represents an at least partly learning effect. As learning effect seems to be present in other types of postural control studies, it is not unreasonable to expect its presence in this study in this way offering an explanation for the moderate ICC values in this study.

In test 3 and 4 an arm movement was added to the postural control task. Again the difference between the two consecutive tests (3 and 4) was the side of the lifted foot. Although postural control test 3 and 4 differ from test 1 and 2 in the addition of the arm movement, no important decrease of the mean values is seen in the first trials. Apparently the learning effect stabilizes after postural control test 1 and 2, and is not influenced by the additional arm movement.

ICC's for the torso angles range from .50 to .93, for the pelvis from .16 to .83. For the mean angle velocities from torso and pelvis they range from .00 to .60. Especially the ICC's for both torso and pelvis movement are substantial to good ("good" means ICC >.80 [23]) in anterior-posterior and in rotation direction. The lateral flexion direction however show lower results. In general the ICC's of the angular velocity are low. This may be due to the fact that it is probably impossible to repeat a postural control correction at the same velocity in two consecutive tests.

Subjects attention during postural control tests is essential [8-17]. Therefore all subjects were blindfolded and tested in a quiet laboratory, to maximise their attention. Nevertheless subjects could still be distracted e.g. immediately after an important disbalance. The influence of concentration on postural control has already been studied, but not in this population.

When comparing the method used in this study with the protocols published in literature, the studies from Cholewicki et al. and Radebold et al. show the largest resemblance [1,7]. However, there is a difference in sitting position on the wobble board. Instead of lifting one foot they placed both feet on a foot support attached to the wobble board. Only the wobble board rested on the force plate. Correction in postural control by the lower extremities was excluded. This made their postural control test more difficult than the one used in this study. We chose a test position with one foot on the ground for safety during the clinical practice. In this way the support foot still influences the postural control, however to learn something about the sensorimotor changes of the lower back we believe that a sitting position is still better than a standing position to evaluate the postural control of the lower back. The duration of each test trial was 7 seconds in Radebolds and Cholewicki's study and 40 seconds in this study. The subjects in the latter study had to perform five test trials. They quantified the repeatability of the CoP parameters by a correlation between two sets of consecutive tests: the first 3 and the last 2. Depending on the CoP parameters different correlation coefficients were found. Some correlation coefficients where high (0.77<R<0.96) others were fair (0.56<R<0.57) and still others were poor (0.14<R<0.40)[7].

## Conclusion

The aim of this study was to investigate the reproducibility of an evaluation method of postural control in an unstable sitting position. The test-retest reproducibility of the procedure is moderate. A learning effect seems to be the explanation. To obtain a good test-retest reproducibility a learning period may be advisable at the beginning of the procedure.

## Abbreviations

LBP: Low Back Pain

CoP: Centre of Pressure

ICC: Intraclass Correlation Coefficient

## Competing interests

The author(s) declare that they have no competing interests.

## Authors' contributions

UVD conceived the study, conducted the research and drafted the manuscript. PV conceived, supervised the study and assisted with the drafting of the manuscript. FH supervised the research procedure, assisted in data acquisition and data management, WD assisted with the statistical analyses and assisted with the correction of the manuscript. BVG supervised the study and assisted with the correction of the manuscript.

## Pre-publication history

The pre-publication history for this paper can be accessed here:



## Figures and Tables

**Table 1:** Test-retest reproducibility (ICC) of four consecutive trials of force plate variables in four different postural control tests.

	**Intraclass Correlation Coefficients**			
	Maximal	Range	Mean	Stdev.

**CoP deviation distance**				
Test 1	0.22	0.22	0.38	0.34
Test 2	0.38	0.38	0.67	0.57
Test 3	0.36	0.36	0.62	0.55
Test 4	0.35	0.35	0.72	0.54
**CoP deviation velocity**				
Test 1	0.11	0.11	0.43	0.34
Test 2	0.30	0.30	0.72	0.52
Test 3	0.36	0.36	0.55	0.43
Test 4	0.29	0.29	0.73	0.56

For each test the ICC of the maximal, range, mean and standard deviation of the CoP deviation distance or velocity are calculated.

**Table 2:** Test-retest intraclass correlation coefficients from the mean angle of torso movement in three directions of four consecutive trials in four different postural control tests.

	**Intraclass Correlation Coefficient**		
**Mean angle deviations torso**	Anteroposterior	Rotation	Lateral

Test 1	0.65	0.80	0.50
Test 2	0.80	0.81	0.67
Test 3	0.77	0.82	0.51
Test 4	0.93	0.81	0.61

**Table 3:** Test-retest intraclass correlation coefficients from the mean angle of pelvis movement in three directions of four consecutive trials in four different postural control tests.

	**Intraclass Correlation Coefficient**		
**Mean angle deviation pelvis**	Anteroposterior	Rotation	Lateral

Test 1	0.66	0.62	0.16
Test 2	0.74	0.43	0.72
Test 3	0.70	0.62	0.31
Test 4	0.83	0.54	0.81

**Table 4:** Test-retest intraclass correlation coefficients from the mean angle velocities of torso movement in three directions of four consecutive trials in four different postural control tests

	**Intraclass Correlation Coefficient**		
**Mean angle velocity torso**	Anteroposterior	Rotation	Lateral

Test 1	0.52	0.46	0.00
Test 2	0.17	0.46	0.48
Test 3	0.28	0.21	0.10
Test 4	0.58	0.25	0.44

**Table 5:** Test-retest intraclass correlation coefficients from the mean angle velocities of pelvis movement in three directions of four consecutive trials in four different postural control tests.

	**Intraclass Correlation Coefficient**		
**Mean angle velocity pelvis**	Anteroposterior	Rotation	Lateral

Test 1	0.28	0.30	0.00
Test 2	0.44	0.41	0.60
Test 3	0.10	0.11	0.06
Test 4	0.41	0.56	0.30
